# Simulation of Spatial Distribution of Multi-Size Bubbles in a Slab Continuous-Casting Mold Water Model

**DOI:** 10.3390/ma16134666

**Published:** 2023-06-28

**Authors:** Yushi Tian, Lijun Xu, Shengtao Qiu, Rong Zhu

**Affiliations:** 1 School of Metallurgical and Ecological Engineering, University of Science and Technology Beijing, Beijing 100083, China; yushi_tian@tom.com (Y.T.);; 2 National Engineering Research Center of Continuous Casting Technology, Central Iron and Steel Research Institute, Beijing 100081, China

**Keywords:** bubbles, collision, breakup, two-phase flow, large eddy simulation, continuous casting mold

## Abstract

In this paper, a fully coupled large eddy simulation model, including the volume of fluid model, the discrete phase model, the bubble-collision model, and the bubble-breakup model was used to simulate the spatial distribution of multi-size bubbles and its impact on the instantaneous two-phase flow in a slab continuous-casting mold. The influence of the bubble-interaction model on the bubbles’ three-dimensional spatial distribution and size distribution, as well as on two-phase flow was discussed. By comparison with the velocity on the meniscus and the average bubble diameter inside a continuous-casting slab water model, the appropriate numerical model was recommended to accurately simulate the two-phase flow and characteristics of discrete bubbles. The submerged entry nozzle and the area around it saw bubble coalescence and breakage more frequently than other areas. The key interaction between the bubbles was their bouncing in the deep region of the mold. In the mold, the average bubble diameter was 0.741 mm, and 44.5% of the total number of bubbles had an approximately average diameter.

## 1. Introduction

**Figure 1:**
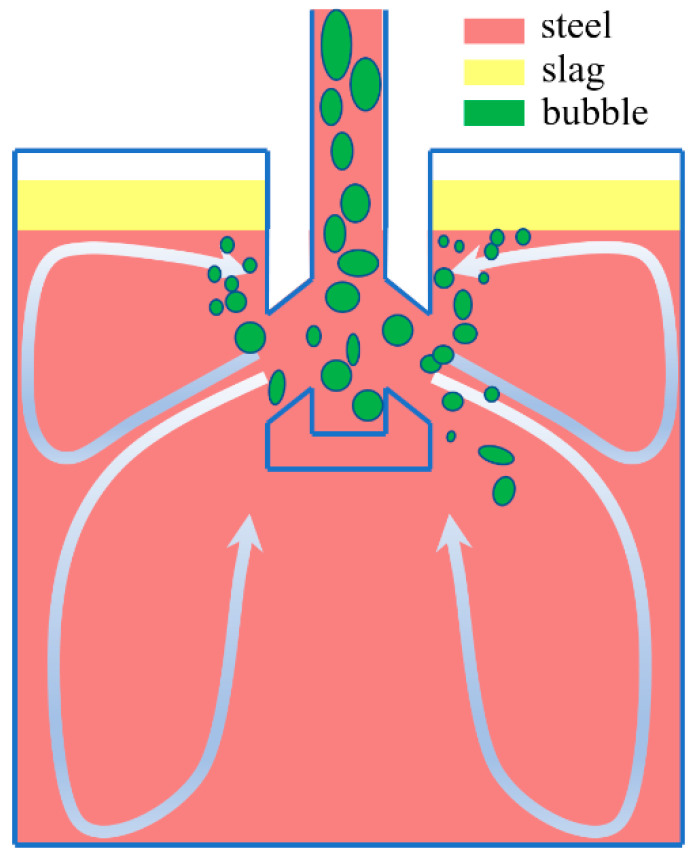
Schematic of multiphase flow in the mold during CC process.

The discrete argon bubbles in a continuous-casting (CC) mold form surface defects after they are entrapped in the solidification front [1]. In addition, the disturbance caused by the bubbles close to the meniscus increases the probability of slag entrainment [2]. However, argon gas injection is still required during the slab CC process as one of the crucial measures to avoid nozzle clogging [3]. Therefore, the current study focuses on improving the multiphase flow (steel, slag, and air) [4] inside the mold after the argon gas injection, as shown in Figure 1. The treatment of argon gas bubbles inside the CC mold is a key and difficult point to accurately calculate the multiphase flow. One widely used method is to treat argon bubbles as the Eulerian phase, similar to molten steel. For example, Liu established a Eulerian–Eulerian (E–E) two-fluid model to investigate the vortex flow in the mold with argon gas injection [5]. Bai employed the E–E multiphase model to simulate turbulent flow with the injection of argon bubbles in a slide-gate tundish nozzle [6]. Using the E–E model, Sánchez-Pérez et al. [7] discussed the distinction between the two-phase flows with the coupled and uncoupled model in a slab mold. Singh [8] developed a three-dimensional E–E mathematical model to study bubble movement in a water model with various parameters. That model successfully predicted the influence of argon gas injection on the multiphase flow but ignored the effect of the change in bubble diameter. Recently, the homogeneous multi-site group (MUSIG) model [9,10] and the non-homogeneous MUSIG model [11] were applied in an E–E approach to include the size distribution of bubbles. The E–E approach extended with the multi-size model was useful in predicting the volume fraction of bubbles and its influence on the flow field. However, it is difficult to calculate the influence on meniscus fluctuation, especially slag entrainment.

Thus, many studies [2,12,13,14,15,16] calculate each argon bubble as a separate individual using a Lagrangian method. Two-way coupled interphase forces were used to actualize the interactions between discrete bubbles and molten steel [17]. Chen et al. systematically studied the bubble distribution and its influence on the flow field using the two-way coupled discrete-phase model (DPM) [2,15,16,18], as well as the entrapment of inclusions using the one-way coupled DPM [19]. The phase interface between the molten steel and the slag phase (or air phase) was determined using the volume of fluid (VOF) model. Wang [20] combined the VOF model, k-ε model, solidification model, and DPM to investigate the impact of the injection gas’s flow rate on the two-phase flow and solidification of steel in a slab CC mold. Yang and Zhang [21,22] used the modified bubble-coalescence-and-breakup model to consider the effect of the bubble diameter based on the above models. When applying DPM to solve the bubble motion, either a Reynolds-averaged Navier–Stokes (RANS) or a large eddy simulation (LES) turbulence model are required for the steel (slag and air) phase. The LES model was recommended to calculate the turbulent flow in a submerged entry nozzle (SEN) and mold due to the presence of asymmetric flow in the mold [23,24]. However, unfortunately, few studies have considered both the collision and the breakup of bubbles and the transient multiphase flow at the same time due to the complexity of the model.

In order to study the spatial and size distribution of bubbles and two-phase flow during the slab CC process, a three-dimensional numerical model including the LES model, VOF model, DPM, bubble-collision model, and bubble-breakup model was constructed. Three bubble-breakup models were compared with the measured bubble diameter and meniscus speed. The main aims of this paper were to propose and establish a mathematical model that considers both the transient multiphase flow inside the CC mold and the coalescence, bounce, and breakup of the bubbles.

## 2. Mathematical Model

### 2.1. Governing Equations

The water and air phases were treated as incompressible Newtonian fluids and the turbulent flow inside the slab CC mold was solved using the LES turbulent model [25]. The water–air-free surface, and the volume fraction of each phase in the mold were determined using the VOF model [26]. The DPM [27,28] was used to calculate the three-dimensional spatial distribution of air bubbles. The interactions between bubbles including the coalescence, breakup, and bounce were included to accurately simulate the interaction between the Eulerian phase and disperse bubbles. The momentum equation is defined as follows:

(1) $$\rho \frac{\partial }{\partial t}\left(u_{i}\right)+\rho \frac{\partial }{\partial x_{j}}\left(u_{i}u_{j}\right)=-\frac{\partial p}{\partial x_{i}}+\frac{\partial }{\partial x_{j}}\left[\left(\mu +\mu_{t}\right)\left(\frac{\partial u_{i}}{\partial x_{j}}+\frac{\partial u_{j}}{\partial x_{i}}\right)\right]+F_{mom,i}$$

where *u* is the speed in m/s. *ρ* is the density in kg/m^3^. *p* is the pressure in Pa. *μ* is the viscosity in kg/(m·s). The subscript *i* and *j* represents the direction *x*, *y*, and *z*. The Smagorinsky–Lilly model [26] was applied in the current study and Equation (2) was used to calculate the turbulent viscosity *μ_t_*. A user-defined function (UDF) was used to achieve *F_mom_*, which was the source term caused by the interaction between the water phase and disperse air bubbles in kg/(m^2^·s^2^).

(2) $$\mu_{t}=\rho L_{s}^{2}\left|\bar{S}\right|$$

where *L_s_* is the mixing length and is defined as $\min\left(\kappa d,C_{s}V^{1/3}\right)$. The *κ* is the Kármán constant and *d* is the closest wall’s distance in m. *S* is the rate-of-strain tensor for the resolved scale in s^−1^. *V* is the cell’s volume in m^3^. *C_S_* is the Smagorinsky constant with a value of 0.1.

The continuity equation is defined as

(3) $$\frac{\partial }{\partial x_{i}}\left(\alpha_{q}u_{q,i}\right)=0$$

where the subscript *q* represents the *q* phase. *α_q_* represents the volume fraction of the *q* phase. *ρ_q_* and ***u_q_*** represent the density in kg/m^3^ and the speed in m/s of the *q* phase, respectively. The density and viscosity of each computational cell are calculated using the mixing law.

The trajectory of each dispersed air bubble is solved using the DPM and the force balance on the bubble is defined as

(4) $$\frac{du_{b}}{dt}=F_{B}+F_{Drag}+F_{Lift}+F_{Pre}+F_{VM}$$

where the ***F_B_***, ***F_Drag_***, ***F_Lift_***, ***F_Pre_***, ***F_VM_*** represent the interphase forces in m/s^2^. ***u_b_*** is the speed of bubbles in m/s. The expression of each interphase force is shown as follows:

(5) $$F_{B}=\frac{\rho_{b}-\rho_{l}}{\rho_{b}}g$$

(6) FDrag=34μCDReρbdb2ul−ub

(7) $$F_{Lift}=C_{L}\frac{\rho_{l}}{\rho_{b}}\left(u_{l}-u_{b}\right)\times \nabla \times u_{l}$$

(8) $$F_{VM}=C_{VM}\frac{\rho_{l}}{\rho_{b}}\left(u_{b}\nabla u_{l}-\frac{du_{b}}{dt}\right)$$

where the *C_D_*, *C_L_*, and *C_VM_* represent the coefficients of drag, lift, and virtual mass, respectively. ***g*** is the acceleration of gravity in m/s^2^. Re is the Reynolds number and is defined as $\frac{\rho_{l}d_{b}\left|u_{l}-u_{b}\right|}{\mu }$. The published studies have shown that these coefficients have a greater impact on interphase force and the specific value, as can be seen in reference [16]. *d_b_* is the bubble diameter in m. The subscripts *l* and *b* represent the liquid phase and disperse bubble, respectively.

The collision of two different bubbles is calculated using the O’Rourke model [29]. The chance of collision is determined in this approach using the idea of a collision volume. The area of the π (*r*_1_ + *r*_2_)^2^ and the covered distance of the smaller bubble in one simulation time step is used to calculate the collision volume, as shown in Equation (9).

(9) $$V_{c}=\pi {\left(r_{1}+r_{2}\right)}^{2}u_{re}\Delta t$$

where *r*_1_ and *r*_2_ represent the radius of two separate bubbles in m. *u_re_* is the relative speed of two separate air bubbles in m/s. ∆*t* is the time step employed in the current calculation in s.

Thus, based on the premise that the bubble has an equal chance of being anywhere in the cell, the definition of the probability of collision is the ratio of collision volume (*V_c_*) and the volume of the computational cell (*V*) where the bubble is located, as shown in Equation (10).

(10) $$P_{c}=\frac{\pi {\left(r_{1}+r_{2}\right)}^{2}u_{re}\Delta t}{V}$$

For *n* small bubbles, the average number of collisions experienced by the large bubble in a time step is defined in Equation (11). In general, the average number in Equation (11) does not correspond to the actual number of collisions. In the O’Rourke model, Equation (12) demonstrates that a Poisson distribution describes the distribution of the probability of the collision number.

(11) $$\bar{N}=\frac{N_{n}\pi {\left(r_{1}+r_{2}\right)}^{2}u_{re}\Delta t}{V}$$

(12) $$P\left(N\right)=e^{-\bar{N}}\frac{{\bar{N}}^{N}}{N!}$$

where *N* is the collision number.

Two bubbles will coalescence or bounce once the collision occurred. Therefore, the collision’s outcome is determined by the collision parameter *b*, defined as $\left(r_{1}+r_{2}\right)\sqrt{Y}$. Coalescence is the outcome of a collision when the real collision parameter *b* is smaller than the critical collision parameter *b_crit_*. If it is not, there will be a bounce. The critical collision parameter *b_crit_* is calculated as follows in the O’Rourke model:

(13) $$b_{crit}=\left(r_{1}+r_{2}\right)\sqrt{\min\left(1.0,\frac{2.4(r_{12}^{3}-2.4r_{12}^{2}+2.7r_{12})}{\mathrm{We}}\right)}$$

(14) $$\mathrm{We}=\frac{\rho u_{re}^{2}\sqrt{d_{1}d_{2}}}{\sigma }$$

where *Y* is the random number with a value between 0 and 1. *r*_12_ is the ratio between the *r*_1_ and *r*_2_. *σ* is the surface tension in N/m. *d*_1_ and *d*_2_ are the diameter of two separate bubbles in m. The velocity of the new bubble after the coalescence is calculated in Equation (15). Also, the velocity of each bubble after the bounce is calculated in Equation (16) according to the basic conservation law.

(15) $$\begin{array}{l} u_{1}^{\prime }=\frac{m_{1}u_{1}+m_{2}u_{2}}{m_{1}+m_{2}}\\ r_{1}^{\prime }={\left(\frac{3\left(m_{1}+m_{2}\right)}{4\pi \rho_{b}}\right)}^{\frac{1}{3}} \end{array}$$

(16) $$\begin{array}{l} u_{1}^{\prime }=\frac{m_{1}u_{1}+m_{2}u_{2}}{m_{1}+m_{2}}+\frac{m_{2}u_{re}}{m_{1}+m_{2}}\left(\frac{b-b_{crit}}{r_{1}+r_{2}-b_{crit}}\right)\\ u_{2}^{\prime }=\frac{m_{1}u_{1}+m_{2}u_{2}}{m_{1}+m_{2}}+\frac{m_{1}u_{re}}{m_{1}+m_{2}}\left(\frac{b-b_{crit}}{r_{1}+r_{2}-b_{crit}}\right) \end{array}$$

where *m* and *u* are the mass and speed of air bubbles in kg and m/s, respectively, and subscripts 1 and 2 represent bubble 1 and bubble 2, respectively.

The turbulent motion of the liquid water causes the air bubble diameter to have the highest critical value. The breakup will happen when the bubble diameter is greater than the critical value. Three breakup models including the Taylor model [30], Kelvin–Helmholtz model [31], and Stochastic model [32] were compared in the current study. In the Taylor model, the oscillation and distortion of a bubble are described by Equation (17). When the oscillation increases to a sufficient level, the bubble will fragment into a number of smaller bubbles.

(17) $$\frac{d^{2}X}{\partial t^{2}}=C_{F}\frac{\rho u_{re}^{2}}{\rho_{b}r}-C_{k}\frac{\sigma }{\rho_{b}r^{3}}X-C_{d}\frac{\mu }{\rho_{b}r^{2}}\frac{dX}{dt}$$

where *X* represents the bubble’s displacement from its center position in m. *C_F_*, *C_k_*, and *C_d_* are the dimensionless constants and are set as 8.0, 5.0, and 1/3, respectively. The bubble is assumed to breakup when *X* > 0.5 *r* and the diameter of the newly formed bubbles is calculated according to the energy balance [33].

The Rayleigh–Taylor instabilities and the Kelvin–Helmholtz waves’ effects on the wave growth on the bubble surface are both considered in the Kelvin–Helmholtz model. Equations (18) and (19) calculate the frequency of the fastest growing wave and the matching wave number on the surface of the bubble, respectively.

(18) $$\Omega_{RT}=\sqrt{\frac{2{\left(g\left(\rho_{l}-\rho_{b}\right)\right)}^{3/2}}{3\sqrt{3\sigma }\left(\rho_{l}+\rho_{b}\right)}}$$

(19) $$K_{RT}=\sqrt{\frac{g\left(\rho_{l}-\rho_{b}\right)}{3\sigma }}$$

Assuming that the bubble is broken up when the Rayleigh–Taylor waves have been growing for a time larger than 0.5/*Ω_RT_*, the radius of the newly formed bubble is calculated as follows:

(20) $$r=\frac{\pi C_{RT}}{K_{RT}}$$

where *C_RT_* is the constant with a value of 0.1.

The Stochastic model assumes that the bubble is subject to breakup when the breakup time is larger than a critical value. The breakup time is defined in Equation (21).

(21) $$t_{b}=B\sqrt{\frac{\rho_{b}}{\rho_{l}}}\frac{r}{\left|u_{re}\right|}$$

where *B* is the constant number of the breakup model and is set as 1.73. The critical breakup time can be calculated using the critical radius as follows:

(22) $$r_{crit}=\frac{{\mathrm{We}}_{\mathrm{crit}}\sigma }{\rho_{l}u_{re}^{2}}$$

We_crit_ represents the critical Weber number, which, in the present investigation, is set as 6. The newly formed bubbles’ diameters are taken as a sample from the analytical Fokker–Planck equation for the probability distribution [32].

### 2.2. Calculation Domain

**Figure 2:**
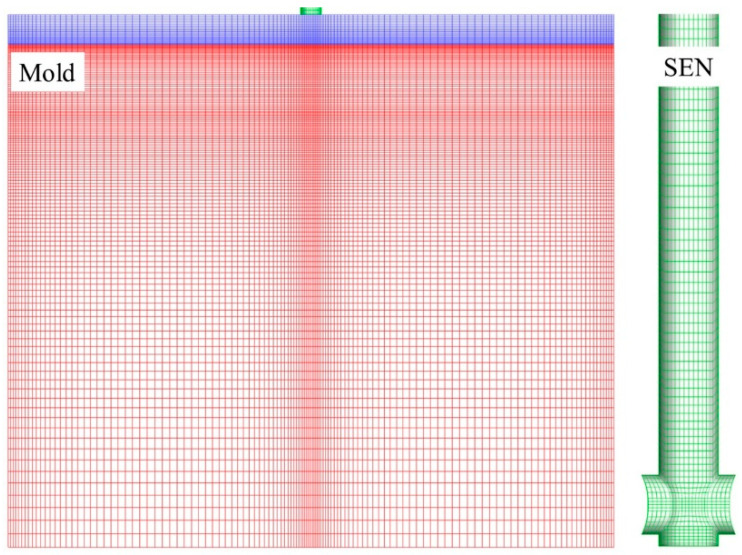
Computational domain and mesh distribution.

The current three-dimensional model was based on a slab CC water model published by Ren and Chen [16,34]. Figure 2 displays the computational domain and mesh distribution. About 0.58 million structured hexahedral meshes were used to divide up the computational domain, and the mesh close to the water–air interface was finetuned. The 510 mm × 50 mm CC mold and the bifurcated nozzle with a 40 mm immersion depth were included. The air phase with a 25 mm initial thickness was taken into consideration at the top. The air bubbles were injected at the SEN inlet. Table 1 provides a summary of the specific parameters that were employed in the present simulation.

### 2.3. Numerical Details

The fully coupled three-dimensional mathematical model was solved by combining commercial ANSYS FLUENT 2021 software and the UDF. The interaction between the liquid water and bubbles was achieved by considering of the interphase forces, and the effect of bubbles passing through the interface or breaking near the interface on the surface level was ignored. The interaction between the bubbles was achieved by the collision and breakup models. The changes in bubble diameter and velocity after the collision and breakup also have a direct impact on the velocity distribution of the liquid water and the surface level. At the SEN input and outflow of the domain, the velocity inlet and pressure outlet boundary conditions were used, respectively. The computation domain’s top surface was intended to be flat and insulated utilizing the free-slip condition. For other walls, the non-slip boundary condition was adopted. The air bubble diameter at the inlet of the SEN was 0.71 mm, determined through the water model [35]. The water–air interface was thought to be where bubbles were eliminated. The escape and reflection conditions were used at the outlet and other walls, respectively. For the pressure–velocity coupling solution, the PISO scheme was employed. The momentum equation was solved using the bounded central differencing scheme, and the equation of the volume fraction was calculated using the compressive method. All variables’ convergence thresholds were set to 10^−4^. The calculation time step of 0.0002 s was chosen to stabilize the calculation because the model was fully coupled and considered bubble coalescence, breakup, and bouncing. Each simulation was run for 130 s.

## 3. Comparison of Bubble Size and Spatial Distribution

### 3.1. Bubble Spatial Distribution

**Figure 3:**
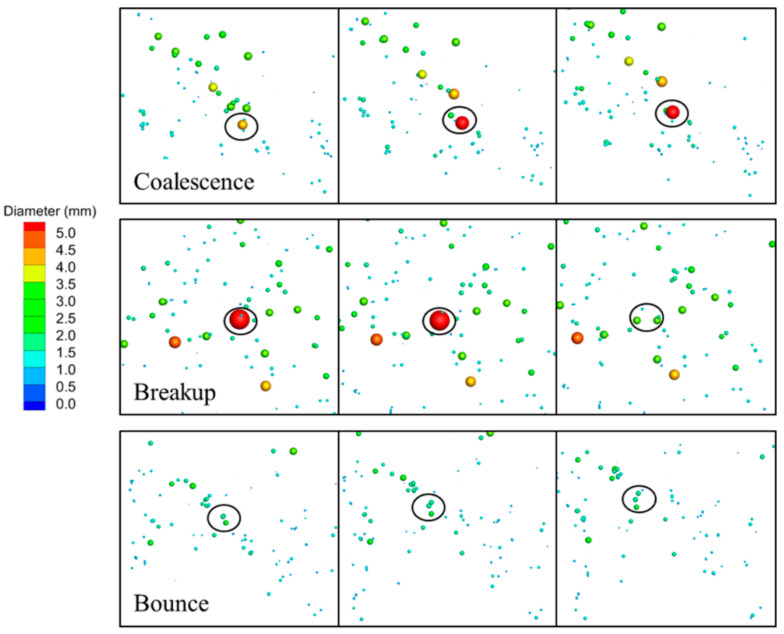
The procedure of the coalescence, breakup, and bounce of air bubbles in the CC mold.

The typical bubble coalescence, breakdown, and bounce process, as predicted by the current model, is seen in Figure 3. The interaction between bubbles, particularly the probability of collision between bubbles, was enhanced by the turbulent transport of the liquid phase inside the SEN and the CC mold. With effective head-on collision, bubble diameter grows with time, and once it reaches the maximum critical diameter, it will disintegrate. The largest diameter of the bubbles that could remain stable at various points along the mold varied. The turbulence flow in the SEN was strong and the bubbles were prone to breakup. The deep section of the mold had weak turbulence flow and a bigger maximum critical bubble diameter. One of the factors contributing to the temporary asymmetric flow in the mold is the SEN’s higher frequency of bubble coalescence and breakup. The outcome of the collision tended to be a bounce if the collision was more oblique, which was the main bubble interaction in the deep part of the CC mold.

**Figure 4:**
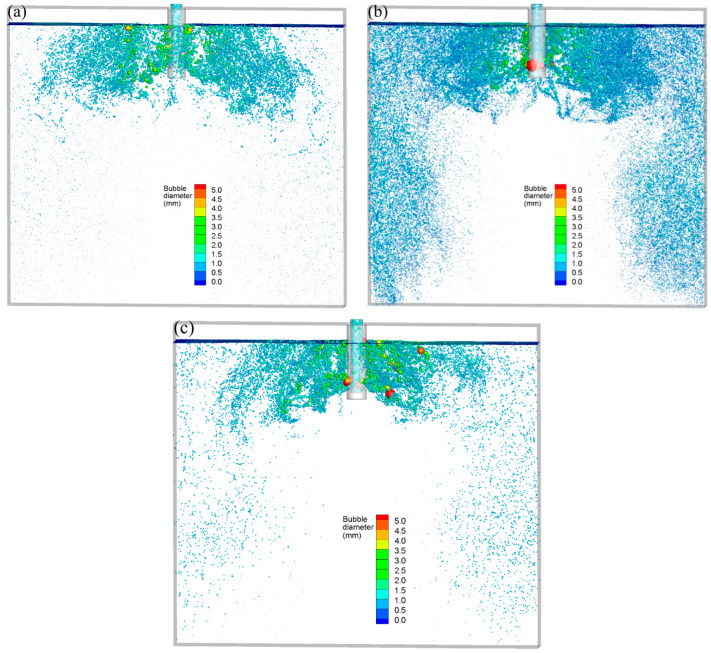
Three-dimensional distribution of spatial of air bubbles at t = 102.0 s: (**a**) Taylor model, (**b**) K-H model, and (**c**) Stochastic model.

In Figure 4, various bubble-breakup models are used to depict the three-dimensional distribution of spatial of air bubbles at t = 102.0 s. The injecting flow rate was set as 90 mL/min, and the casting speed was set as 0.425 m/min. It can be seen that air bubbles were almost full of the entire mold in three bubble-breakup models. The lower recirculation flow carried the fine newly formed bubbles that broke off from the giant bubbles to the deep part of the CC mold. However, due to the different breakup mechanisms, the distribution of bubble diameter showed an obvious distinction with the Taylor model, K-H model, and Stochastic model.

**Figure 5:**
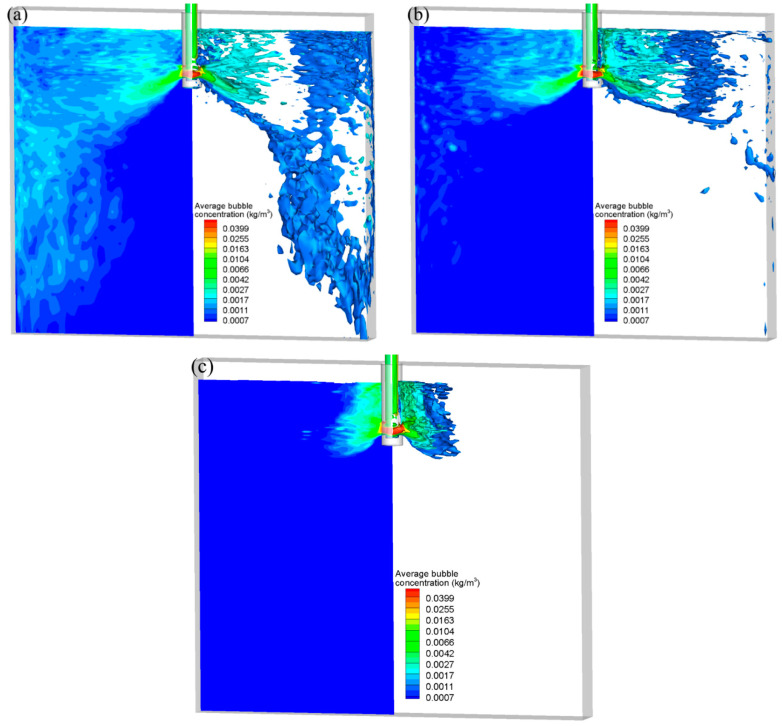
Comparison of bubbles’ average mass concentration with different bubble-breakup models: (**a**) Taylor model, (**b**) K-H model, and (**c**) Stochastic model.

The effect of the bubble-breakup model on the three-dimensional average bubble mass concentration was compared in Figure 5. The contour of the bubble concentration is shown on the left, and the bubble concentration ISO surface is shown on the right. The time-averaged results were calculated for 30 s after the two-phase flow attained a stable state. It can be seen that from the Taylor model, K-H model and Stochastic model, the bubbles’ degree of dispersion inside the CC mold increased gradually. Most of the bubbles were concentrated near the SEN with the Stochastic breakup model. The spatial distribution of bubbles with the Stochastic model in Figure 4c shows that there are many fine bubbles located in the deep part of the CC mold. However, the average bubble concentration in the mold deep region was almost zero in Figure 5c. This indicates that the number and size of fine air bubbles generated by bubble breakup with the Stochastic model were between the Taylor model and K-H model.

**Figure 6:**
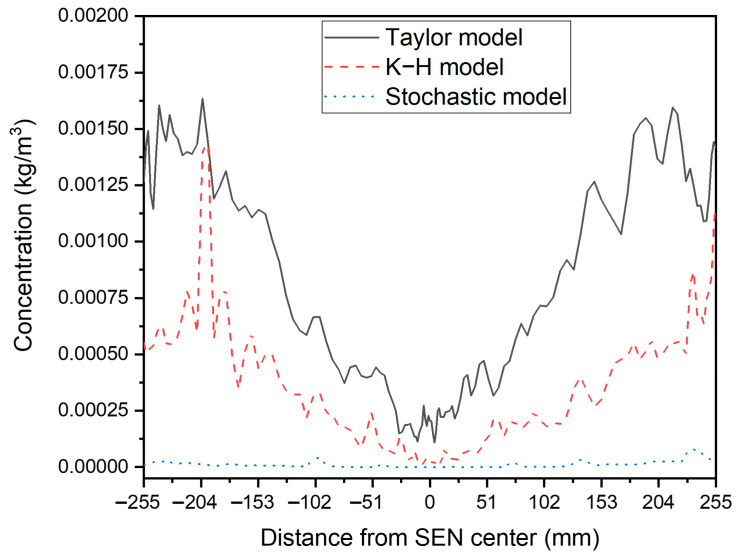
Effect of the breakup model on bubble average concentration at 170 mm below the meniscus.

**Figure 7:**
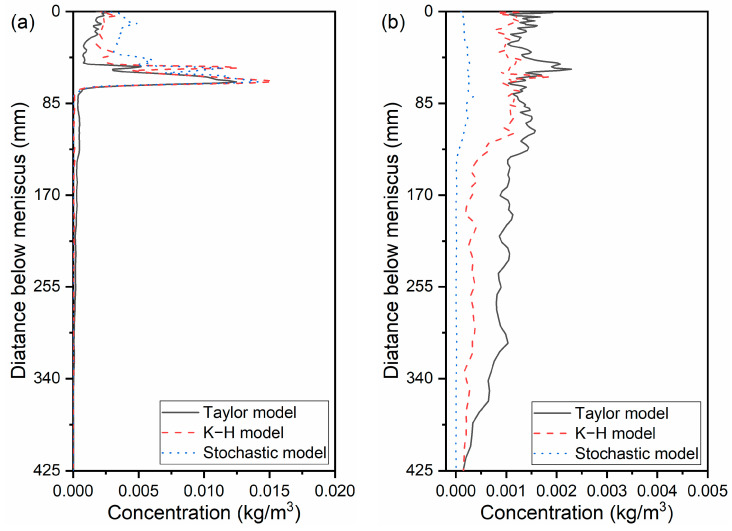

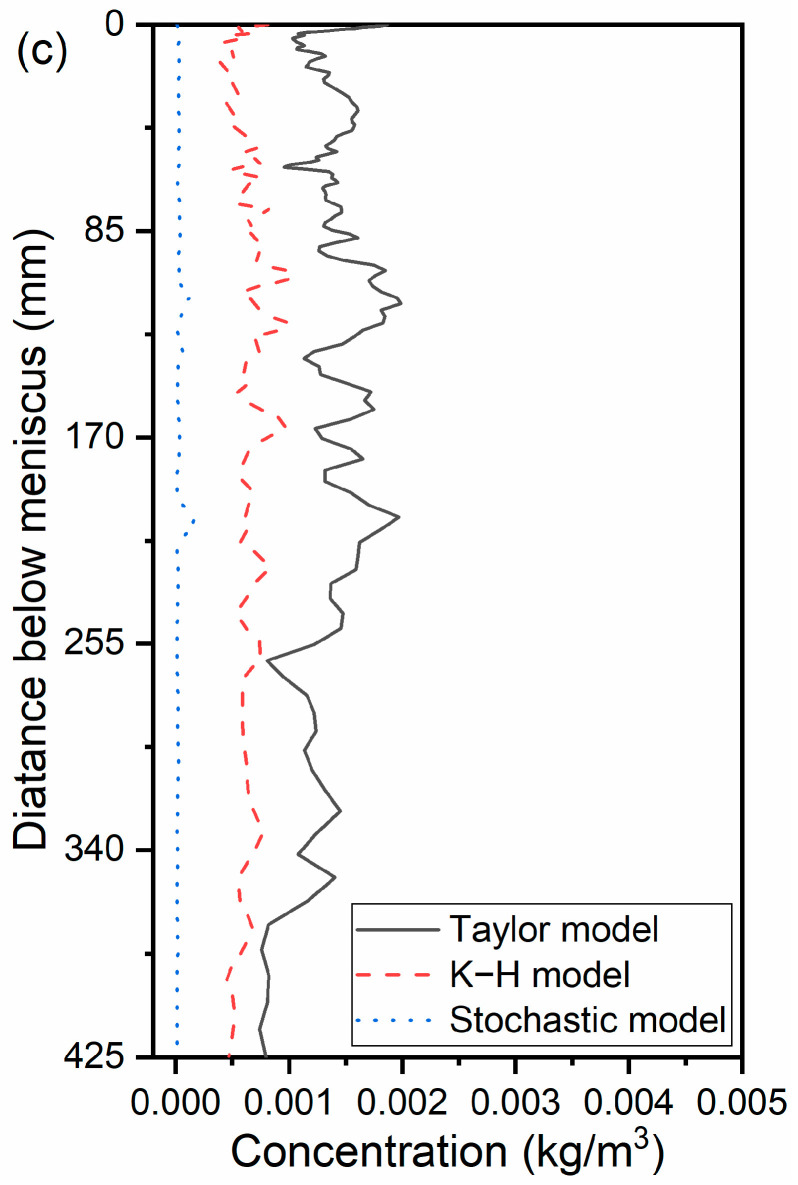
Mass concentration of air bubbles along the casting direction: (**a**) near the SEN, (**b**) 1/4 width of the CC mold, and (**c**) near the narrow face.

The effect of the bubble-breakup model on the average bubble concentration at 170 mm below the meniscus was compared in Figure 6. The bubble concentration with the Taylor model was significantly higher than that with the K-H model and Stochastic model. The bubble concentration was close to zero in the Stochastic model. The bubble concentration increased gradually from the mold center to the narrow face at the lower part of the mold. Because of the small buoyance of fine bubbles, they were more likely to move with the molten steel. Thus, the number of fine bubbles was higher near the narrow face at the deep part of the CC mold. The bubble concentration decreased from the SEN to the narrow face at the upper part of the mold due to the rise of the large bubbles. Figure 7 analyses the mass concentration of air bubbles along the casting direction quantitatively in more detail. From the Taylor model to the K-H model to the Stochastic model, the mass concentration of air bubbles in the vicinity of the SEN steadily rose. The average bubble concentration did, however, steadily decline close to the 1/4 mold width and narrow face. The biggest bubble concentration was found close to the SEN, and it was almost ten times closer to the narrow face.

### 3.2. Distribution of Bubble Diameter

**Figure 8:**
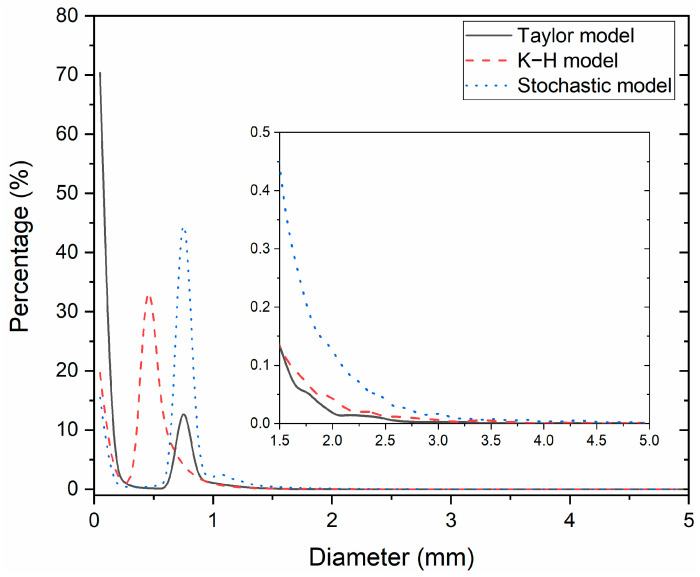
Effect of the bubble-breakup model on the diameter of bubbles in the CC mold.

The impact of the bubble-breakup model on the distribution of the bubble diameter is depicted in Figure 8. With the Taylor breakup model, the percentage of bubbles less than 0.25 mm reached 70%, whereas the percentage in the K-H model and the Stochastic model was between 15% and 20%. Another diameter peak under the Taylor model was 0.75 mm and reached 12.6%. For the K-H model and Stochastic model, the peak bubble diameter and number proportion was 0.45 mm and 33.3%, and 0.75 mm and 44.5%, respectively. Compared with the Taylor model and K-H model, the Stochastic model had the largest number proportion of bubbles with a 1–2 mm diameter. The number proportion of bubbles greater than 2 mm was very small.

**Figure 9:**
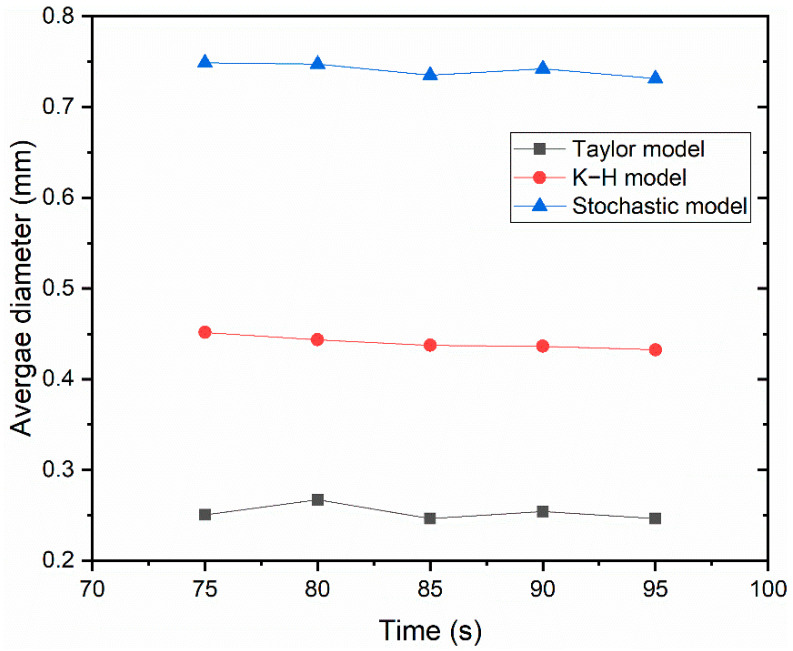
Variation of the diameter with time.

**Figure 10:**
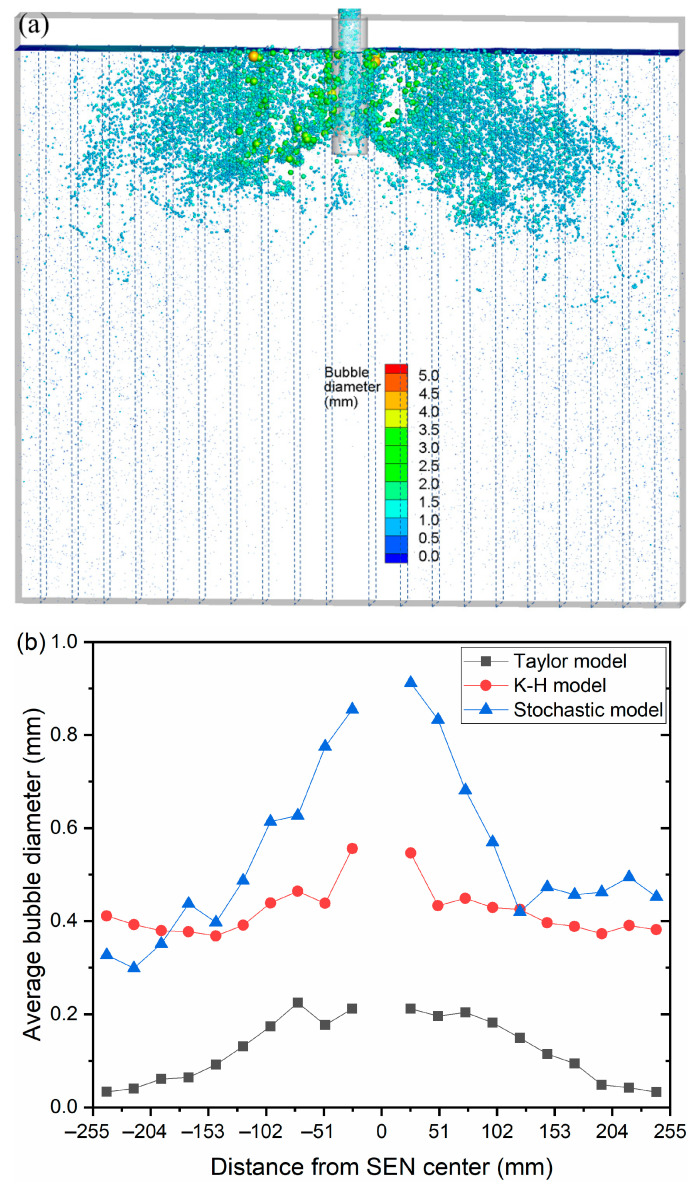
Effect of the bubble-breakup model on the average bubble diameter at different regions: (**a**) statistical interval, (**b**) distribution of the average bubble diameter.

The bubble’s average diameter with respect to time is shown in Figure 9. As can be observed, the bubble’s average diameter changed slightly with time under the current transient simulation and remained basically unchanged after the flow reached a stable state. The average bubble diameter in the entire mold was 0.253 mm, 0.440 mm, and 0.741 mm with the Taylor model, K-H model, and Stochastic model, respectively. The average bubble diameter obtained by the Stochastic model was in the best agreement with the measured values. The area between the narrow face and the SEN was divided into 10 equal zones, as indicated in Figure 10, and the bubble average diameter in each zone was computed. Due to their high buoyancy, the large size bubbles produced by the bubble coalescence climbed to the meniscus swiftly. Thus, from the SEN to the mold edge, the average bubble diameter dropped. With the Stochastic model, the bubble’s average diameter was 0.85 mm close to the SEN and 0.4 mm close to the narrow face.

### 3.3. Removal Position of Bubbles

**Figure 11:**
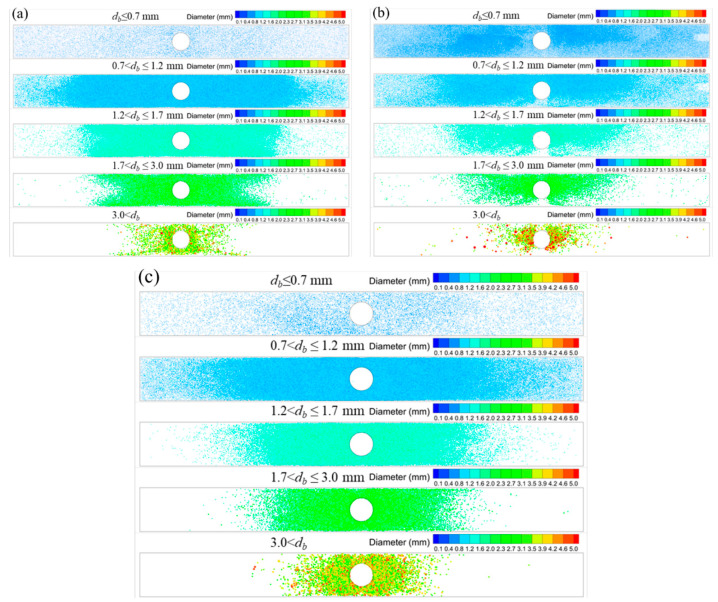
Effect of the bubble-breakup model on the removal position at the meniscus: (**a**) Taylor model, (**b**) K-H model, and (**c**) Stochastic model.

In order to compare the bubble removal position at the meniscus, the position was recorded and outputted in real-time during the calculation. The removal position for differently sized bubbles and breakup models is shown in Figure 11. It is clear that from the removal position, the horizontal penetration distance of bubbles less than 1.7 mm can span the full width of the mold. Bubbles larger than 3.0 mm had a horizontal penetration distance that did not surpass half the mold’s width. With the K-H model, the removal position for bubbles larger than 3.0 mm was discovered close to the narrow face. This can be brought on by the big bubbles that coalesced in the deep part of the mold (Figure 3b).

## 4. Comparison of the Instantaneous Flow Field

**Figure 12:**
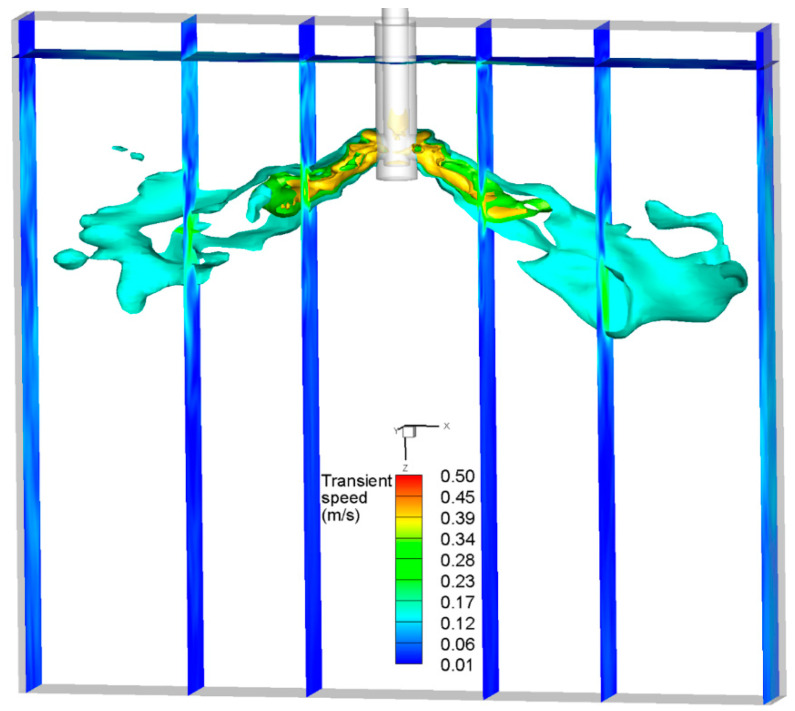
Distribution of the instantaneous flow filed at a certain moment using the Stochastic breakup model.

The stochastic breakup model is employed to depict the instantaneous two-phase flow in Figure 12 at a specific time. The air injection rate was 90 mL/min, and the casting speed was 0.425 m/min. The flow field in the mold had an instantaneous asymmetrical distribution. At this time, there was a region with a large velocity near the meniscus on the right of the mold, resulting in fluctuations in the speed and liquid level of the meniscus significantly greater than those of the meniscus on the left of the mold. This asymmetric flow was caused by the collision and bounce of bubbles with the bottom of the SEN and the characteristics of turbulence. Moreover, the asymmetric distribution was exacerbated and even dominated by the coalescence, breakup, and bounce of bubbles, especially at the bottom of the SEN and the meniscus.

**Figure 13:**
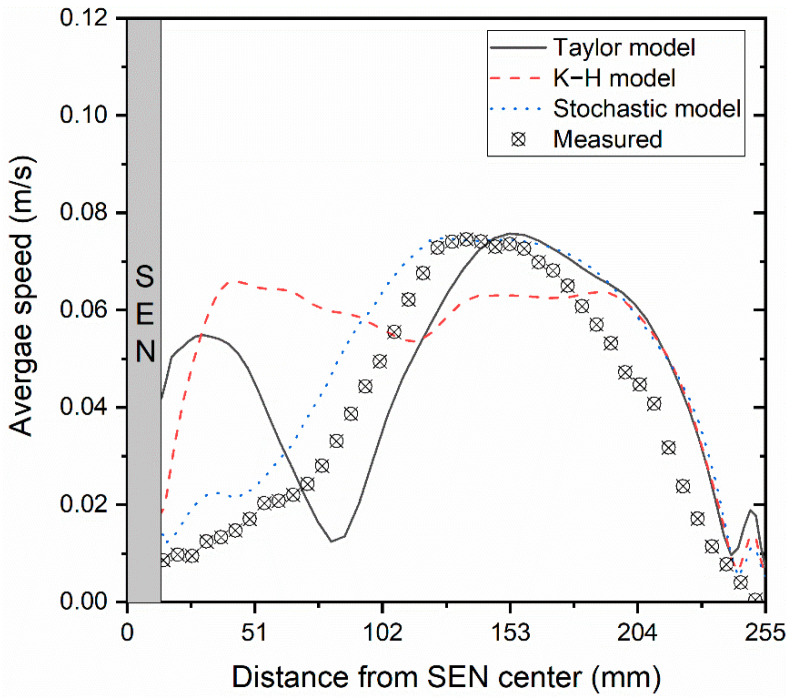
Comparison of the predicted and measured [35] average speed along the center line of the meniscus.

To quantitatively analyze how various bubble-breakup models affect the flow field, Figure 13 compares the predicted average speed and the measured velocity [35] at the center line of the meniscus. It can be seen that the speed predicted by the Stochastic breakup model is in the best agreement with the measured results, which is relative to the average bubble diameter distribution shown in Figure 9. The bubble-breakup model had a great influence on the speed distribution since the bubble-breakup model directly determined the bubble diameter and then influenced the distribution of the speed through the liquid–bubble interaction.

**Figure 14:**
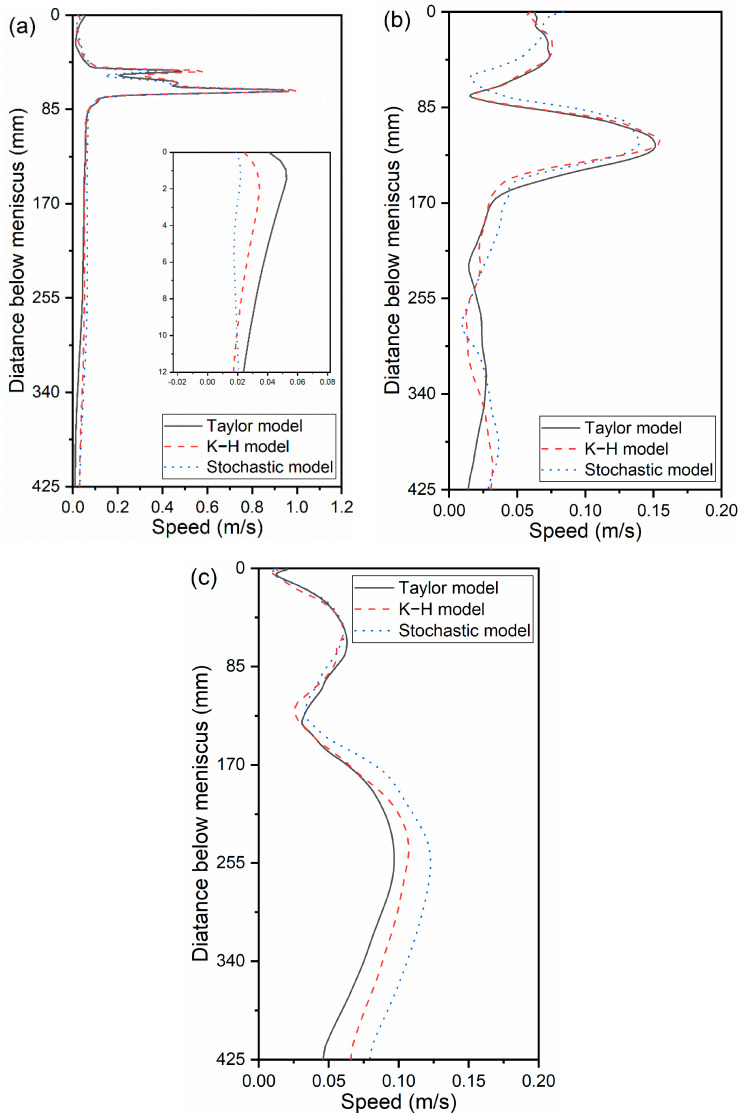
Distribution of the average speed along the casting direction: (**a**) 5 mm from the SEN, (**b**) 1/4 width of the CC mold, and (**c**) 5 mm from the narrow face.

Figure 14 compares the distribution of average speed along the casting direction. The findings indicate that the area between the upper SEN outlet and the meniscus was where the bubble-breakup models had the greatest impact on the speed near the SEN. Figure 14a demonstrates how the speed within 12 mm of the meniscus gradually decreased using the Taylor, K-H, and Stochastic models. This is contrary to the bubble diameter distribution in Figure 10b. The effect of the bubble on the lowering of velocity is greater the larger the bubble’s diameter. It should be noted that the speed of liquid steel will increase when the bubble diameter rises to a certain point owing to the bubble’s floating.

## 5. Conclusions

This work suggests a validated numerical model that includes the LES model, VOF model, DPM, bubble-collision model, and bubble-breakup model. It was explained how the bubble-breakup model affected the spacing and size of dispersed bubbles and two-phase flow in the CC mold. The following is a succinct summary of the conclusions:

1. The instantaneous asymmetrical distribution of bubbles and two-phase flow in a slab CC mold was successfully predicted. The two-way coupled DPM took into account the bubbles’ coalescence, breakup, and bounce.
2. The distribution of the bubble diameter showed an obvious distinction between the different bubble-breakup models. The average bubble concentration near the SEN gradually increased from the Taylor model to K-H model and Stochastic model. However, the average bubble concentration was gradually increased from the 1/4 width of the mold to the narrow face.
3. The predicted average bubble diameter in the entire mold was 0.253 mm, 0.440 mm, and 0.741 mm with the Taylor model, K-H model, and Stochastic model, respectively. The proportion of fine bubbles was overpredicted with the Taylor and K-H breakup model. The bubble-breakup model had a noticeable impact on the distribution of the speed due to the direct determination of the size distribution of bubbles.
4. The Stochastic breakup model had the best agreement with the measured data when compared to the average bubble diameter and meniscus speed. Thus, the fully coupled LES model, VOF model, and DPM with the collision and breakup model is recommended to correctly calculate the two-phase flow and distribution of multi-size bubbles during the CC process.

## Figures and Tables

**Table 1:** Parameters of the simulation.

Parameter	Value
Total length	562 mm
Cross size	510 mm × 50 mm
SEN outlet angle	0°
SEN immersion depth	40 mm
Casting speed	0.425 m/min
Air flow rate	90 mL/min
Density of water	1000 kg/m^3^
Viscosity of water	0.001 kg·m^−1^·s^−1^
Density of air	1.225 kg/m^3^
Viscosity of air	1.789 × 10^−5^ kg·m^−1^·s^−1^
Surface tension	0.07197 N/m

## Data Availability

Not applicable.
